# Vancomycin-Induced Leukocytoclastic Vasculitis: A Rare Case Report

**DOI:** 10.1177/2324709618820873

**Published:** 2018-12-21

**Authors:** Pratibha Sharma, Eliza Sharma, Sanjay P. Neupane, Suyash Dahal, Sumit Dahal

**Affiliations:** 1 Maimonides Medical Center, Brooklyn, NY, USA; 2 New York Presbyterian Hospital-Weill Cornell Medical Center, New York, NY, USA; 3 KIST Medical College and Teaching Hospital, Lalitpur, Nepal; 4 St. Joseph Hospital, Bangor, ME, USA

**Keywords:** vancomycin, vasculitis, leukocytoclastic vasculitis

## Abstract

Vancomycin causes different types of hypersensitivity reactions, ranging from localized skin reactions to generalized cardiovascular collapse. However, cases of vancomycin-induced leukocytoclastic vasculitis are rare. In this article, we present a case where the patient developed palpable purpura on his bilateral lower limbs following treatment with vancomycin. He was diagnosed with vancomycin-induced leukocytoclastic vasculitis that resolved without sequelae after withdrawal of vancomycin.

## Introduction

Vancomycin has been in use since 1954, and it is widely used for the treatment of serious infections caused by methicillin-resistant *Staphylococcal aureus* (MRSA) or in individuals who have failed, cannot tolerate, or are allergic to other antibiotics. With the increasing resistance to antibiotics, the use of vancomycin is expected to increase. This has resulted in greater focus and accumulation of new data related to the drug’s safety profile. Vancomycin, mainly in its parenteral form, has been attributed to cause different types of hypersensitivity reactions, ranging from localized skin reactions to generalized cardiovascular collapse.^1-4^ Cases of vancomycin-induced leukocytoclastic vasculitis (LV) have, however, only rarely been reported.^5-9^

## Case Report

Our patient was an 83-year-old male with past medical history of hypertension, hyperlipidemia, atrial fibrillation, cerebrovascular accident, and end-stage renal disease. He was on scheduled hemodialysis treatment through a catheter on his right chest. He was brought to the emergency department for fever and rigors of 1-day duration, with a recorded body temperature of 101.8°F. There was no cough, chest pain, shortness of breath, abdominal pain, diarrhea, vomiting, headache, altered mental status, and pain or burning on urination. Physical examination and the initial investigations failed to elucidate any obvious focus of infection. The patient was admitted for possible sepsis associated with infected dialysis catheter, and treated empirically with intravenous cefepime 1 g every 24 hours, intravenous vancomycin 15 mg/kg body weight every 24 hours, and intravenous metronidazole 500 mg every 8 hours. The dose of vancomycin was altered as needed to maintain the vancomycin trough level between 15 µg/mL and 20 µg/mL. Two sets of initial blood culture and subsequent dialysis catheter tip culture grew *Staphylococcus aureus* resistant to methicillin but sensitive to vancomycin, daptomycin, rifampin, and tetracycline, while a transthoracic echocardiography revealed right atrium mass, suggestive of endocarditis. The patient was therefore continued only on intravenous vancomycin for MRSA endocarditis.

**Figure 1.**
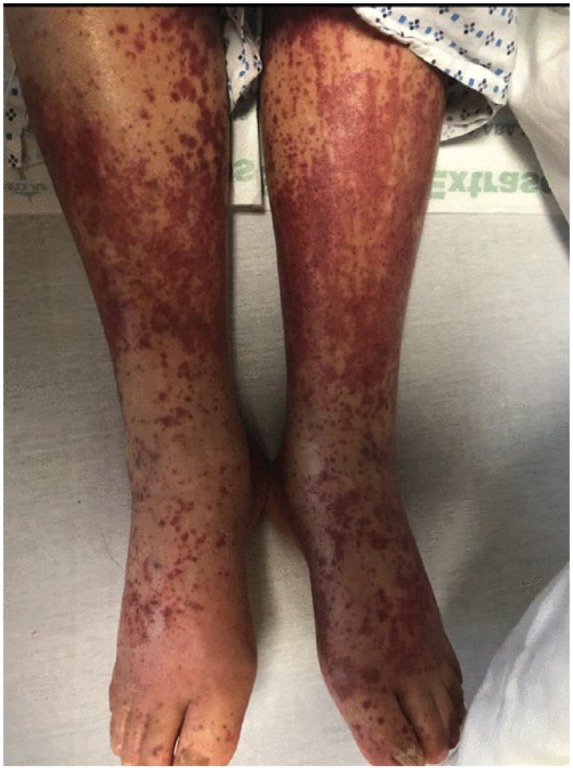
The palpable purpura on bilateral lower limbs.

**Figure 2.**
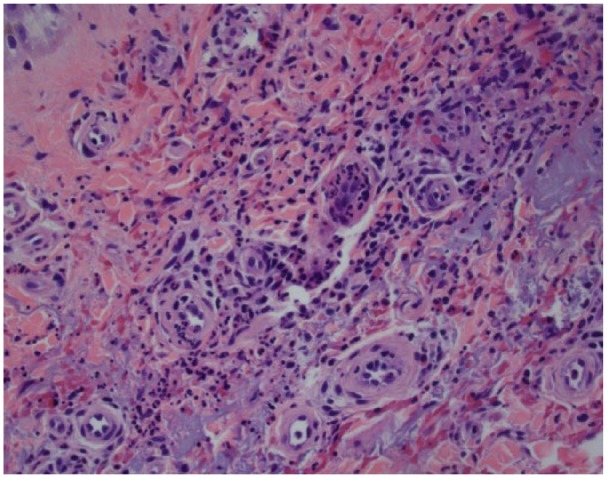
Skin biopsy showing perivascular neutrophilic infiltrate and fibrinoid necrosis of vessel wall.

On the 11th day of admission, the patient developed palpable purpura on his both lower limbs (Figure 1). He denied any pain or itching. While the erythrocyte sedimentation rate was elevated to 45 mm/h (normal = 0-30 mm/h), rest of his immunological workup including c-ANCA, p-ANCA, and atypical ANCA were negative, and C3, C4 levels were normal. Skin biopsy was done, which revealed severe leukocytoclastic necrotizing small cell vasculitis consistent with hypersensitivity vasculitis related to drug therapy (Figure 2). So his vancomycin was switched to daptomycin. The purpura started to resolve within 3 days of discontinuing vancomycin. Both his vasculitis and initial clinical condition continued to resolve during the rest of his hospitalization, and he was successfully discharged after 22 days of hospital stay.

## Discussion

The most common cutaneous reaction with vancomycin, termed “red man syndrome,” is an idiopathic infusion reaction related to immunoglobulin E–mediated mast cell degranulation and not a true allergic reaction.^10^ Vancomycin, however, also causes different hypersensitivity reactions ranging from skin rashes and eruptions to severe anaphylaxis.^1-4^

LV is a small vessel vasculitis, and it is thought to result from the deposition of circulating immune complexes into vessel walls activating the complement pathway. LV is usually limited to the skin and may manifest as palpable purpura, maculopapular rash, bullae, papules, nodules, or ulcers. The different causes of LV include drugs, infections, malignancies, and connective tissue disorders. While the commonly attributed drugs include β-lactam antibiotics, nonsteroidal anti-inflammatory drugs, and diuretics, few cases of vancomycin-induced LV have been reported before.^5-9^

A review of the published literature shows that the onset of vancomycin-induced LV can be highly variable, ranging from within 24 hours after drug initiation to as late as 1 month after administration. It is not dose related, and case of vasculitis after a single dose has been reported. Our patient developed vasculitis on the 11th day of vancomycin and is within the expected range of developing these reactions. As with our patient, most cases manifested as palpable purpura or maculopapular rash appearing first on the lower limbs. These lesions have been reported to spread to mid-thigh, abdomen, and chest as well. Most cases of vancomycin-induced LV are self-limited and resolve with withdrawal of vancomycin without any sequelae. While oral corticosteroids have been used for vancomycin-induced LV, their efficacy has not been established. The time to recovery is variable, ranging from days to weeks. Our patient had all the clinical and histological hallmarks of LV. Alternative causes for LV were pursued in our patient. His immunological workups were negative. While he had initially received cefepime and metronidazole, they had long been discontinued when he developed the rashes and were less likely to cause vasculitis. Systemic bacterial infection with MRSA cannot be completely ruled out as the cause for LV in our patient. However, the temporal association of starting vancomycin with the appearance of the purpura and of withdrawing vancomycin with the resolution of the purpura as well as the typical appearance and location of the rash consistent with prior reports of vancomycin-associated LV pointed to vancomycin as the most plausible cause for the LV. This was further supported by the use of the Naranjo adverse drug reaction probability scale, which indicated that the likelihood of vancomycin being the cause of the vasculitis was probable with a score of 5.^11^

Clinicians worldwide need to be aware of this rare hypersensitivity reaction with vancomycin administration. This is all the more relevant as the use of vancomycin, and with it the incidence of its rare adverse effects, increases in the coming days.
